# Food and nutrient intake in relation to mental wellbeing

**DOI:** 10.1186/1475-2891-3-14

**Published:** 2004-09-13

**Authors:** Reeta Hakkarainen, Timo Partonen, Jari Haukka, Jarmo Virtamo, Demetrius Albanes, Jouko Lönnqvist

**Affiliations:** 1 Department of Mental Health and Alcohol Research, National Public Health Institute, Helsinki, Finland; 2 Department of Epidemiology and Health Promotion, National Public Health Institute, Helsinki, Finland; 3 Division of Cancer Epidemiology and Genetics, National Cancer Institute, Bethesda, MD, USA

**Keywords:** anxiety, depression, food, insomnia, nutrition

## Abstract

**Background:**

We studied food consumption and nutrient intake in subjects with depressed mood, anxiety and insomnia as indices of compromised mental wellbeing.

**Methods:**

The study population consisted of 29,133 male smokers aged 50 to 69 years who entered the Alpha-Tocopherol, Beta-Carotene Cancer Prevention Study in 1985–1988. This was a placebo-controlled trial to test whether supplementation with alpha-tocopherol or beta-carotene prevents lung cancer. At baseline 27,111 men completed a diet history questionnaire from which food and alcohol consumption and nutrient intake were calculated. The questionnaire on background and medical history included three symptoms on mental wellbeing, anxiety, depression and insomnia experienced in the past four months.

**Results:**

Energy intake was higher in men who reported anxiety or depressed mood, and those reporting any such symptoms consumed more alcohol. Subjects reporting anxiety or depressed mood had higher intake of omega-3 fatty acids and omega-6 fatty acids.

**Conclusions:**

Our findings conflict with the previous reports of beneficial effects of omega-3 fatty acids on mood.

## Background

Diet has an effect on mood and cognitive function [1]. There is some evidence that deficiency or supplementation of nutrients can affect not only mood, but also behavioral patterns.

A double-blind placebo-controlled trial with 30 patients showed that omega-3 essential fatty acid supplements alleviated symptoms in patients with bipolar disorder [2]. In a recent double-blind, placebo-controlled trial on 231 young adult prisoners, by comparing the number of their disciplinary offences before and during the supplementation, antisocial behavior was reduced by the supplementation of vitamins, minerals and essential fatty acids [3]. Vitamin D supplementation during winter was reported to improve mood in a double-blind, placebo-controlled trial on 44 healthy volunteers [4].

A number of studies have shown that acute tryptophan depletion produces depressive symptoms and results in worsening of mood [5]. Folic acid deficiency may also correlate with depression, and it has particular effects on mood, cognitive as well as social functioning [6]. Recently, it have been reported that low levels of dietary folic acid are associated with elevated depressive symptoms in middle-aged men [7].

In general, a low-fat diet may have negative effects on mood [8], and altered dietary fat intake can lead to acute behavioral effects such as drowsiness, independent of energy consumption [9]. A high intake of proteins also seems to increase alertness [1]. Increased dietary serine and lysine may be linked to the pathogenesis of major depressive disorder [10]. Apart from specific nutrients or vitamins, certain foods may have an effect on mental wellbeing. Warm milk, for instance, has been traditionally used as self-medication for insomnia. Individuals drinking regular coffee with caffeine have reported to have decreased total sleep time and sleep quality, and increased sleep latency [11]. It has been reported that people with a high consumption of fish appear to have a lower prevalence of major depressive disorder [12,13]. Recently, it has been also reported that increased fish intake in people without depressive symptoms had no substantial effect on mood [14].

Depressed subjects tend to consume more carbohydrates in their diets than non-depressed individuals [15], and they show heightened preference for sweet carbohydrate or fat rich foods during depressive episodes [16]. High carbohydrate intakes increase brain uptake of tryptophan, which in turn stimulates the synthesis of serotonin [1]. At present, there are some studies focusing on the use of dietary supplements in individuals with mental disorders, but there is a lack of consistent data concerning the impact of nutrition, diet and eating habits on mental health.

## Aims

We set out to study whether food consumption and intake of nutrients in subjects with depressed mood, anxiety and insomnia differed from those in subjects without any such symptoms.

## Methods

This study was based on the cohort of a randomized, double-blind, placebo-controlled primary prevention trial testing the hypothesis that daily supplementation with α-tocopherol or β-carotene reduces the incidence of lung and other cancers [17]. The study participants were recruited between 1985 and 1988 from the total male population 50–69 years of age, residing in southwestern Finland (n = 290,406). These men were sent a questionnaire on current smoking status and willingness to participate in the trial. Smokers of at least five cigarettes per day and who were willing to participate were then invited to visit their local study center for further evaluation of their eligibility. A previous cancer diagnosis, current severe angina with exertion, chronic renal insufficiency, cirrhosis of the liver, alcohol dependence, or a disorder limiting participation in the long-term trial, such as mental disorder or physical disability, were reasons for exclusion. A total of 29,133 men were randomly assigned to receive supplements of either α-tocopherol, β-carotene, both, or placebo, in a 2 × 2 factorial design. The ethics review boards of the participating institutions approved the study, and all subjects provided written informed consent prior to randomization.

At baseline, subjects completed a questionnaire on their background and medical history, including three questions on mental wellbeing. These items concerned anxiety, depressed mood and insomnia experienced in the past four months. Height and weight were also measured, and a blood sample was drawn for determining total and high-density lipoprotein (HDL) cholesterol concentrations. Diet and alcohol consumption was assessed from a self-administered dietary history questionnaire [18], which asked the frequency of consumption and the usual portion size of 276 food items during the past year, using a color picture booklet as a guide for portion size. Complete dietary data were available for 27,111 participants.

Dietary nutrient data were analyzed by linking the questionnaire data to the food composition database of the National Public Health Institute, Finland. For analysis, we considered three main groups: principal nutrients, specific nutrients selected on the basis of a priori hypotheses, and certain foods. The principal nutrients were energy, carbohydrates, proteins and fats. The hypothesis-based nutrients were omega-3 and omega-6 fatty acids, lysine, serine, tryptophan, and two vitamins, vitamin D and folic acid. Omega-3 fatty acids from fish consist of long-chain fatty acids, while the omega-3 fatty acids in vegetables are shorter-chain molecules. The food items included were fish, milk, meat, vegetables, margarine, coffee and alcohol. We also evaluated the total energy intake.

The trial involved three follow-up visits annually. At each follow-up visit the participants were asked whether they had felt anxiety, depression, or insomnia since the preceding visit (Have you felt feelings of depression in last three months? Have you felt feelings of anxiety in last three months? Have you had insomnia in last three months?). To identify subjects who suffered chronically from these symptoms we took into account the symptoms reported throughout the first follow-up year, i.e. at baseline and the three follow-up visits (at baseline, 4 months, 8 months and 12 months). Men reporting anxiety, depression, insomnia, or all these symptoms at all four visits were included in these analyses.

### Statistics

As potential risk factors, baseline age, body-mass index (BMI), energy intake, alcohol consumption, education level, marital status and smoking were entered into regression models as covariates. Dietary factors were adjusted for energy intake in the models [19].

## Results

At study entry, 4314 (16%) men reported depressed mood in the past four months, 6498 (24%) feelings of anxiety, and 5550 (21%) insomnia. The mean intake of energy was 1 to 3% greater and consumption of alcohol 30 to 33% greater in subjects with any such symptoms, compared with symptom-free individuals (Table 1). Men reporting all three symptoms consumed as much as 47% more alcohol than those without any symptoms. Subjects with insomnia consumed 7% less coffee than symptom-free individuals, whereas those with depressed mood or anxiety consumed only about 2% less coffee (Table 2).

**Table 1:** Baseline characteristics of subjects with self-reported depressed mood, anxiety or insomnia, and subjects with all three or none of the symptoms.

	Depressed mood		Anxiety		Insomnia		All three symptoms		No symptoms	
	(n = 4314)		(n = 6498)		(n = 5550)		(n = 1670)		(n = 19116)	
	Mean	SD	Mean	SD	Mean	SD	Mean	SD	Mean	SD
Age (years)	57.2	4.9	57.0	4.8	57.8	5.1	56.9	4.8	57.8	5.1
Energy (kcal/day)	2877	813	2888	801	2828	818	2886	863	2793	777
Alcohol consumption (g/day)	21.7	26.2	21.5	25.1	22.0	25.4	24.3	28.5	16.5	19.8
BMI (kg/m^2^)	26.3	3.9	26.2	3.9	26.1	3.9	26.1	3.8	26.3	3.7
Total serum cholesterol (mmol/l)	6.16	1.19	6.22	1.18	6.15	1.19	6.13	1.21	6.26	1.16
Serum HDL-cholesterol (mmol/l)	1.24	0.36	1.26	0.36	1.27	0.37	1.27	0.38	1.23	0.34

**Table 2:** Baseline daily food consumption and nutrient intake of subjects self-reporting depression, anxiety or insomnia, and all three or none of the symptoms.

	Depressed mood (n = 4314)		Anxiety (n = 6498)		Insomnia (n = 5550)		All three symptoms (n = 1670)		No symptoms (n = 19116)	
	Mean	SD	Mean	SD	Mean	SD	Mean	SD	Mean	SD
Fish (g)	39.3	30.2	39.9	30.2	40.1	30.3	40.3	32.9	39.3	29.8
Milk (g)	212	315	203	316	226	321	219	325	220	322
Coffee (ml)	595	374	601	372	567	364	583	382	609	349
Meat (g)	78.0	38.4	80.2	38.8	77.6	38.6	78.0	37.8	78.6	37.2
Vegetables (g)	256	103	264	104	253	104	255	106	263	101
Margarine (g)	11.5	21.1	11.5	20.8	10.7	20.3	11.8	21.3	10.2	19.8
Carbohydrate (g)	308	97.7	309	96.8	300	96.7	304	97.6	303	94.0
Protein (g)	105	30.6	105	30.2	103	31.2	105	31.6	103	28.9
Fat (g)	125	41.8	125	41.8	123	42.2	125	43.4	122	40.6
Sugar (g)	38.5	27.5	38.3	28.0	36.9	26.7	37.7	27.6	38.1	26.5
Lysine (g)	6.42	1.97	6.44	1.95	6.37	2.01	6.44	2.04	6.30	1.86
Serine (g)	4.12	1.31	4.27	1.30	4.22	1.33	4.28	1.35	4.18	1.24
Tryptophan (g)	1.28	0.38	1.29	0.38	1.27	0.39	1.29	0.40	1.26	0.36
Omega-3 fatty acids (total) (g)	2.21	0.93	2.24	0.92	2.16	0.92	2.23	0.97	2.14	0.87
Omega-3 fatty acids (from fish) (g)	0.47	0.28	0.48	0.29	0.48	2.89	0.49	0.30	0.46	0.28
Omega-3 fatty acids (from vegetables) (g)	1.77	0.82	1.79	0.80	1.70	0.80	1.77	0.86	1.70	0.77
Omega-6 fatty acids (g)	10.12	6.82	10.14	6.70	9.70	6.65	10.17	7.05	9.44	6.31
Omega-6/omega-3 ratio	4.47	2.00	4.45	2.01	4.41	2.03	4.50	2.30	4.34	1.85
Folic acid (μg)	342	106	344	105	335	106	340	107	336	103
Vitamin D (μg)	5.59	3.21	5.65	3.18	5.60	3.18	5.72	3.23	5.45	3.08

In subjects with depressed mood, the mean intake of omega-6 fatty acids was 7% greater than in symptom-free subjects. In individuals with anxiety, the mean intake of omega-6 fatty acids was 7% greater and that of omega-3 fatty acids from vegetables 5% greater than in subjects with no symptoms. Intake of fish or omega-3 fatty acids from fish were not associated with anxiety or depressed mood.

When the symptoms reported during the first trial follow-up year were taken into analysis, 782 men reported depressed mood, 1237 feelings of anxiety, 1234 insomnia, and 166 men all three symptoms on all four occasions. The mean intake of energy was 7% greater in subjects reporting all three symptoms repeatedly compared with symptom-free individuals. Subjects with insomnia consumed 11% less coffee but 10% more milk than those with no insomnia. Both in subjects with depressed mood and with anxiety, the mean intake of total omega-3 fatty acids was 9% greater and that of omega-3 fatty acids from vegetables 6% greater than in respective symptom-free subjects, whereas the mean intake of omega-6 fatty acids was 6% greater in subjects with depressed mood and 9% greater in subjects with anxiety.

## Discussion

Our subjects reporting anxiety had higher intakes of omega-3 and omega-6 fatty acids, but omega-3 fatty acids from fish were not linked to anxiety. Margarine was the main source of both omega-3 fatty acids from vegetables and omega-6 fatty acids. Subjects with depressed mood also had a higher intake of omega-6 fatty acids. Because 3138 (73%) subjects with depressed mood also had feelings of anxiety, it may be that anxiety is the dominant symptom, and the greater intake of omega-3 and omega-6 fatty acids is primarily related to feelings of anxiety.

Previously, it has been suggested that omega-3 fatty acids may alleviate the effects of depressive symptoms but not those of mania [20]. Recently, we have reported that the low dietary intake of omega-3 fatty acids is not associated with depression [21]. Our present results show now that individuals suffering from symptoms of depressed mood have higher intakes of omega-6 and omega-3 fatty acids. More investigation is needed to elucidate the specific effects of omega-3 fatty acids on mood.

Subjects with any or all of the symptoms consumed more alcohol than the symptom-free subjects. Subjects with all three symptoms consumed most alcohol of all, and they received 6% of their total energy from alcohol, compared with 4% in subjects with no symptoms. Energy from alcohol, however, did not explain the differences in the mean intake of energy between groups. Body-mass index was lower, despite a higher caloric intake, in subjects with any of the symptoms compared with symptom-free subjects.

Subjects reporting insomnia drank more milk than symptom-free subjects, but less coffee. Warm milk has long been taken as a self-medication for insomnia, and our finding among those with insomnia accords with this traditional habit. In addition, they avoided consuming large amounts of coffee, which is known to have impact of sleep. We also found that subjects reporting depressed mood consumed more carbohydrates than subjects with no symptoms. This finding is consistent with the attempt by depressed subjects to alleviate the carbohydrate craving associated with symptoms of depression.

Tryptophan intake showed no association with mental wellbeing in our study population. Interestingly, a number of negative studies has been published recently, suggesting that the effects of tryptophan depletion on mood are inconsistent [22-24], and the rationale for augmentation has now been challenged [25]. The intakes of vitamin D and folic acid exceeded the daily recommendations and showed no association with mental wellbeing. Neither did the consumption of fish, milk, meat or vegetables.

## Limitations

There are some limitations in our study. Our study was a cross-sectional study, and it cannot provide causal evidence on the association between the diet and symptoms of depression, anxiety or insomnia. The study participants included only men, aged 50 to 69 years, and all were smokers. Our exclusion criteria limit the generalization of our findings, but the study still provides valid and reliable data on a community-based, homogenous sample of older men.

Dietary intake and alcohol consumption were assessed with a validated food use questionnaire to measure the habitual dietary intake over the previous year as completely as possible. For most nutrients, both the reproducibility and the validity of this method are 0.6 to 0.7 [18]. For example, they are 0.66 and 0.73 for energy intake, 0.88 and 0.85 for alcohol, 0.70 and 0.75 for carbohydrates, and 0.70 and 0.64 for vitamin D, respectively.

The assessment of self-reported depression was based on a single item only that might have compromised the specificity, but not sensitivity. For example, two questions only may be as effective as more detailed screening instruments in detecting probable cases of major depression [26]. One of these questions ("During the past month, have you often been bothered by feeling down, depressed, or hopeless?") is rather similar to the item that we applied for being indicative of depressed mood.

## Conclusion

The scientific examination of relationships between nutrition and mental wellbeing is a relatively new area of study. Most of the studies focus on the use of dietary supplements, which provide more concentrated amounts of specific nutrients than most food sources. There are few data evaluating food consumption and nutrient intake among subjects with compromised mental health. Our main finding was that we did not find any association between omega-3 fatty acids from fish and mental wellbeing. In general, more attention need to be paid to the intake of nutrients in patients suffering from symptoms of depression, anxiety or insomnia. Further studies are needed to clarify complex associations between the diet and mental wellbeing, and to elucidate their mechanisms of action.
